# Co-Overexpression of *OsNAR2.1* and *OsNRT2.3a* Increased Agronomic Nitrogen Use Efficiency in Transgenic Rice Plants

**DOI:** 10.3389/fpls.2020.01245

**Published:** 2020-08-12

**Authors:** Jingguang Chen, Xiaoqin Liu, Shuhua Liu, Xiaoru Fan, Limei Zhao, Miaoquan Song, Xiaorong Fan, Guohua Xu

**Affiliations:** ^1^ State Key Laboratory of Crop Genetics and Germplasm Enhancement, MOA Key Laboratory of Plant Nutrition and Fertilization in Low-Middle Reaches of the Yangtze River, Nanjing Agricultural University, Nanjing, China; ^2^ CAAS-IRRI Joint Laboratory for Genomics-Assisted Germplasm Enhancement, Agricultural Genomics Institute in Shenzhen, Chinese Academy of Agricultural Sciences, Shenzhen, China; ^3^ Department of Agronomy, Purdue University, West Lafayette, IN, United States

**Keywords:** *Oryza sativa*, *OsNAR2.1*, *OsNRT2.3a*, co-overexpression, agronomic nitrogen use efficiency, nitrogen recovery efficiency

## Abstract

The NO_3_^-^ transporter plays an important role in rice nitrogen acquisition and nitrogen-use efficiency. Our previous studies have shown that the high affinity systems for nitrate uptake in rice is mediated by a two-component NRT2/NAR2 transport system. In this study, transgenic plants were successful developed by overexpression of *OsNAR2.1* alone, *OsNRT2.3a* alone and co-overexpression of *OsNAR2.1* and *OsNRT2.3a*. Our field experiments indicated that transgenic lines expressing *p35S:OsNAR2.1* or *p35S:OsNAR2.1-p35S:OsNRT2.3a* constructs exhibited increased grain yields of approximately 14.1% and 24.6% compared with wild-type (cv. Wuyunjing 7, WT) plants, and the agricultural nitrogen use efficiency increased by 15.8% and 28.6%, respectively. Compared with WT, the ^15^N influx in roots of *p35S:OsNAR2.1* and *p35S: OsNAR2.1-p35S:OsNRT2.3a* lines increased 18.9%‑27.8% in response to 0.2 mM, 2.5 mM ^15^NO_3_^–^, and 1.25 mM ^15^NH_4_^15^NO_3_, while there was no significant difference between *p35S:OsNAR2.1* and *p35S:OsNAR2.1-p35S:OsNRT2.3a* lines; only the ^15^N distribution ratio of shoot to root for *p35S:OsNAR2.1-p35S:OsNRT2.3a* lines increased significantly. However, there were no significant differences in nitrogen use efficiency, ^15^N influx in roots and the yield between the *p35S:NRT2.3a* transgenic lines and WT. This study indicated that co-overexpression of *OsNAR2.1* and *OsNRT2.3a* could increase rice yield and nitrogen use efficiency.

## Introduction

Nitrogen (N) is an essential macronutrient for plant growth and crop productivity, it affects all levels of plant function from metabolism to resource allocation, growth, and development (Crawford, 1995; Scheible et al., 2004). Nitrate nitrogen (NO_3_^–^) and ammonium nitrogen (NH_4_^+^) are two main inorganic nitrogen sources in plant growth. Conventionally cultivated rice is submerged, and nitrification is inhibited, NH_4_^+^ is the main inorganic nitrogen in rhizosphere soil, therefore, rice is generally considered as ammonium-preferring plant (Arth et al., 1998; Kronzucker et al., 1999). However, for its well-developed aerial tissue, rice can transport and secrete oxygen from photosynthesis above ground to the rhizosphere (Aurelio et al., 2003). Oxygen can stimulate the growth and reproduction of nitrifying bacteria in the rhizosphere, so the part of NH_4_^+^ in the rhizosphere can be nitrified into NO_3_^–^ (Li et al., 2008). Therefore, in the actual growth of rice, its root system has been in the mixed nutrition of NH_4_^+^ and NO_3_^–^ (Kirk and Kronzucker, 2005). Aurelio et al. (2003) found that most rice varieties could absorb the same amount of NH_4_^+^ and NO_3_^–^, and the amount of NO_3_^–^ absorbed by rice would be much larger than that of NH_4_^+^ because of the strong nitrification in rhizosphere. During the late stage of rice growth and development, when it has been going through the process of long-term alternate wetting and drying irrigation or the whole growth stage of upland rice, the uptake of NO_3_^–^ was also higher than that of NH_4_^+^ by rice (Arth et al., 1998; Wang et al., 2004).

In addition to be a nutrient, NO_3_^–^ also serves as a signaling molecule, which induces changes in plant growth and gene expression (Kirk and Kronzucker, 2005; Liu et al., 2015). For example, NO_3_^–^ could break seed dormancy (Alboresi et al., 2005), and induced multiple genes for plant growth and development (O’Brien et al., 2016). Furthermore, the development of lateral root, leaf growth and altering flowering time could be regulated by NO_3_^–^(Zhang et al., 1999; Rahayu et al., 2005; Castro et al., 2011; Hsu and Tsay, 2013; Naz et al., 2019). NO_3_^–^ could be sensed by the plant, and the uptake of NO_3_^–^, especially in rice, can increase the uptake of NH_4_^+^ to a certain extent (Duan et al., 2006; Chen et al., 2017).

Two different NO_3_^–^ uptake systems in plants, the high-affinity NO_3_^–^ uptake systems (HATS) and low-affinity NO_3_^–^ uptake systems (LATS) are regulated by NO_3_^–^ supply and enable plants to cope with low or high NO_3_^–^ concentrations in soils (Miller et al., 2007; Xu et al., 2012). As we known, the NRT2 and NPF families contribute to HATS and LATS responding the NO_3_^–^ uptake and translocation in plants (Miller et al., 2007; Léran et al., 2014). Some high-affinity NO_3_^–^ transporters belonging to the NRT2 family have been reported to require a partner protein, NAR2, for their function (Tong et al., 2005; Yong et al., 2010; Xu et al., 2012). In rice, two-component NRT2-NAR2 system also exists in the NO_3_^–^ transport process, and OsNAR2.1 is the essential partner of OsNRT2 nitrate transporters for nitrate uptake over low and high concentration range (Miller et al., 2007; Feng et al., 2011; Liu et al., 2015). OsNRT2.1, OsNRT2.2, and OsNRT2.3a were similarly shown to require OsNAR2.1 for NO_3_^–^ uptake, and their interaction at the protein level was demonstrated by using a yeast two hybrid assay, an oocyte expression system and western blotting (Feng et al., 2011; Yan et al., 2011; Liu et al., 2014).

Previous studies have shown that *pOsNAR2.1:OsNAR2.1* expression or *pOsNAR2.1:OsNAR2.1* expression increased the expression of *OsNRT2.1* and *OsNAR2.1*, and *OsNRT2.1* or *OsNAR2.1* driven by *OsNAR2.1* promote can improve nitrate uptake and final grain yield in rice (Chen et al., 2016; Chen et al., 2017). Yan et al. (2011) reported that knockdown of *OsNAR2*.1 could suppress the expression of *OsNRT2.1, OsNRT2.2*, and *OsNRT2.3a* in rice roots, and then reduced nitrate uptake and affected its growth. OsNRT2.1 and OsNRT2.2 are responsible for NO_3_^−^ uptake from the soil (Katayama et al., 2009; Fan et al., 2017). OsNRT2.3a is a root-to-shoot NO_3_^−^ transporter (Tang et al., 2012). However, overexpression of *OsNRT2.3a* alone did not increase nitrogen uptake and transport in rice (Fan et al., 2016). In this study, we focus on co-overexpressed *OsNAR2.1* and *OsNRT2.3a* in rice, overexpression of *OsNAR2.1* or *OsNRT2.3a* alone as control and explored the effect of co-overexpression of *OsNAR2.1* and *OsNRT2.3a* on NO_3_^–^ uptake, yield and nitrogen use efficiency uptake by rice.

## Materials and Methods

### Construction of Vectors and Rice Transformation

The open reading frames (ORF) of *OsNAR2.1* (AP004023) and *OsNRT2.3a* (AK109776) were amplified from the full-length cDNA of rice cv. Nipponbare, and the primer sequences were shown in **Supplementary Table S1**. PrimeSTAR HS DNA Polymerase (TaKaRa Biotechnology Co., Ltd, Dalian, China) was used to amplify the ORF of *OsNAR2.1* and *OsNRT2.3a*, and the two-step PCR parameters were 95 °C for 5 min followed by 98 °C for 10 s, 68 °C for 2 min (30 cycles) and 72 °C for 10 min. Full-length coding sequences of *OsNAR2.1* was amplified subcloned into the pSAT4.35SP vector. After PI-PspI digestion of pSAT4.35SP-OsNAR2.1, the fragment was cloned into the PI-PspI site of vector pRCS2-ocs-nptII (Goodin et al., 2007) in the antisense orientation and got OsNAR2.1-pRCS2-ocs-nptII, as *p35S:OsNAR2.1*, then confirmed by gene sequencing. The ORF of *OsNRT2.3a* was subcloned into the pSAT6.supP vector. After I-SceI digestion of pSAT6.supP-*OsNRT2.3a*, the fragment was cloned into the I-SceI site of vector pRCS2-ocs-nptII and got *NRT2.3a*-pRCS2-ocs-nptII, as *p35S:OsNRT2.3a*, confirmed by gene sequencing. For the construction of co-overexpression of *OsNAR2.1* and *OsNRT2.3a* (*p35S:OsNAR2.1-p35S:OsNRT2.3a*), we used I-SceI for digestion of *NRT2.3a*-pRCS2-ocs-nptII construction, the fragment was cloned into the I-SceI site of construction NAR2.1-pRCS2-ocs-nptII. The vectors were introduced into *Agrobacterium tumefaciens* strain EHA105 by electroporation and then transformed into rice as described previously (Tang et al., 2012).

### qRT-PCR and Southern Blot Analysis

The total RNA extraction of plants and genes expression analysis were performed as described previously (Chen et al., 2016; Chen et al., 2017). The primers for PCR are shown in **Table S2**.

The Southern blot was used to identify the copy number of inserted T-DNA. The genomic DNA extraction of the T2 plant, DNA digestion and hybridization were following the reported paper (Chen et al., 2017).

### Field Experiments for Harvest Yield

We selected the positive T1 transgenic lines by PCR. The sequence of the resistance screening gene (792 bp) on the vector was amplified by forward primer (5’ ATGATTGAACAAGATGGATTGCA 3’) and reverse primer (5’ GAAGAACTCGTCAAGAAGGCGAT 3’). The Southern blot was used to identify the copy number of inserted T-DNA of the T2 plant, we chose the single-copy T2 lines and planted them to screen T3 and T4 generation plants with stable inheritance. T1, T2, and T4 generation plants were grown in plots at the Nanjing Agricultural University from June to October in 2013, 2014, and 2016 in Nanjing, Jiangsu. The rice plants of T3 generation plants were cultivated in plots at the Experimental Site of Zhejiang University June to October 2015 in Changxing, Zhejiang. Soil properties in Nanjing field experiment was described as before (Chen et al., 2016; Chen et al., 2017). Basal applications of 30 kg P/ha as Ca(H2PO4)2 and 60 kg/K ha (KCl) were made to all plots 3 days before transplanting. N fertilizer as urea accounted for 40%, 30% and 40% of the total N fertilizer was applied prior to transplanting, at tillering, just before the heading stage, respectively.

T1‑T2 generation transgenic plants and wild-type (cv. Wuyunjing 7, WT) plants were planted in the plots with 300 kg N/ha. T3 generation transgenic plants and WT plants were planted in 3 plots with 150 kg N/ha and the plots without nitrogen fertilizer as blank control. T4 generation transgenic plants and WT plants were planted in 3 plots with 300 kg N/ha and the plots without nitrogen fertilizer as blank control. T2 generation transgenic plants of cv. Nipponbare and wild-type (cv. Nipponbare, NP) plants were planted in 3 plots with 150 kg N/ha and the plots without nitrogen fertilizer as blank control. The plots size was 2 × 2 m, and the seedlings were planted in a 10 × 10 array. Compared with WT, the flowering period of *p35S:OsNAR2.1* lines were 4‑5 days earlier, that of *p35S:OsNRT2.3a* lines had no significant change, and that of *p35S:OsNAR2.1-p35S:OsNRT2.3a* lines were 2‑3 days earlier. During rice flowering and mature stages, the samples of T4 generation transgenic plants and WT were collected for further analysis.

### Dry Weight, Total Nitrogen Measurement, and Calculation of Nitrogen Use Efficiency

We conducted biomass and nitrogen analysis of the T4 generation shoot samples grown under 300 kg N/ha fertilizer condition according to our previous reported method (Chen et al., 2016). Dry matter at anthesis stage (DMA), grain yield (GY), dry matter at maturity stage (DMM), total nitrogen accumulation at anthesis stage (TNAA), grain nitrogen accumulation at maturity stage (GNAM), total nitrogen accumulation at maturity stage (TNAM), harvest index (HI), the contribution of pre-anthesis assimilates to grain yield (CPAGY), dry matter translocation (DMT), dry matter translocation efficiency (DMTE), contribution of pre-anthesis nitrogen to grain nitrogen accumulation (CPNGN), nitrogen translocation (NT), nitrogen translocation efficiency (NTE), nitrogen harvest index (NHI), agronomic nitrogen use efficiency (ANUE), physiological nitrogen use efficiency (PNUE), and nitrogen recovery efficiency (NRE) were calculated according to Chen et al. (2016; 2017). CPAGY (%) = (DMT/GY) × 100%; HI (%) = (GY/DMM) × 100%; DMT (kg/ha) = DMA – (DMM – GY); DMTE (%) = (DMT/DMA) × 100%; CPNGN (%) = (NT/GNAM) × 100%; NT (kg/ha) = TNAA – (TNAM – GNAM); NTE (%) = (NT/TNAA) × 100%; ANUE (kg/kg) = (GY – GY of zero-N plot)/N supply; PNUE (kg/kg) = (GY – GY of zero-N plot)/TNAM; NRE (%) = (TNAM –TNAM of zero-N plot)/N supply.

### Determination of Total N Content, Root ^15^N Influx Rate, and ^15^N Distribution Ratio

WT and transgenic rice seedlings were grown in IRRI solution containing 1 mM NH_4_^+^ for 2 weeks, and then transferred into three different forms of nitrogen treatments including in 0.2 mM NO_3_^–^, 2.5 mM NO_3_^–^, and 1.25 mM NH_4_NO_3_. The full strength IRRI solution used has the following compositions: 1.0 mM MgSO4·7H2O, 1.25 mM NH4NO3, 0.3 mM KH2PO4, 1.0 mM CaCl2, 0.35 mM K2SO4, 0.5 mM Na2SiO3, 20.0 μM Fe-EDTA, 20.0 μM H3BO3, 9.0 μM MnCl2, 0.77 μM ZnSO4, 0.32 μM CuSO4, and 0.39 μM (NH4)6Mo7O24, pH 5.5. The nutrient solution was replaced every 3 days and the pH was adjusted to 5.5 every day. Plants were grown in a growth room with a 14 h light (30°C) (8:00‑22:00)/10 h dark (22°C) (22:00‑8:00) and 60% relative humidity. After 3-week treatment, the biomass and nitrogen concentration of every line under each N treatment were measured.

For root ^15^N uptake experiment, rice seedlings were grown in 1mM NH_4_^+^ for 3 weeks and then under nitrogen starvation condition for 1 week before ^15^N uptake experiment. The plants were rinsed in 0.1 mM CaSO_4_ for 1 min, then transferred to a solution containing either 0.2 mM ^15^NO_3_^–^, 2.5 mM ^15^NO_3_^–^ or 1.25 mM ^15^NH_4_^15^NO_3_ (atom % ^15^N: 99%) for 5 min, and finally rinsed again in 0.1 mM CaSO_4_ for 1 min.

For root ^15^N distribution ratio experiment, WT and transgenic seedlings were grown in 1 mM NH_4_^+^ for 3 weeks, nitrogen starvation was then carried out for 1 week. The plants were rinsed in 0.1 mM CaSO_4_ for 1 min, then transferred to the nutrient solution containing 0.2 mM ^15^NO_3_^–^, or 1.25 mM ^15^NH_4_^15^NO_3_ for 12 h, and finally rinsed again in 0.1 mM CaSO_4_ for 1 min. The ^15^N influx rate and ^15^N distribution ratio were calculated following the method reported in Chen et al. (2017) and Tang et al. (2012).

### Statistical Analysis

For our data statistical analysis, the single factor analysis of variance (ANOVA) and Tukey’s test data analysis was applied. All statistical evaluations were conducted using the IBM SPSS Statistics ver. 20 software. (SPSS Inc., Chicago, IL, United States).

## Results

### Generation of Transgenic Plants Expressing *p35S:OsNAR2.1-p35S:OsNRT2.3a* Construction and Field Traits Analysis

**Figure 1:**
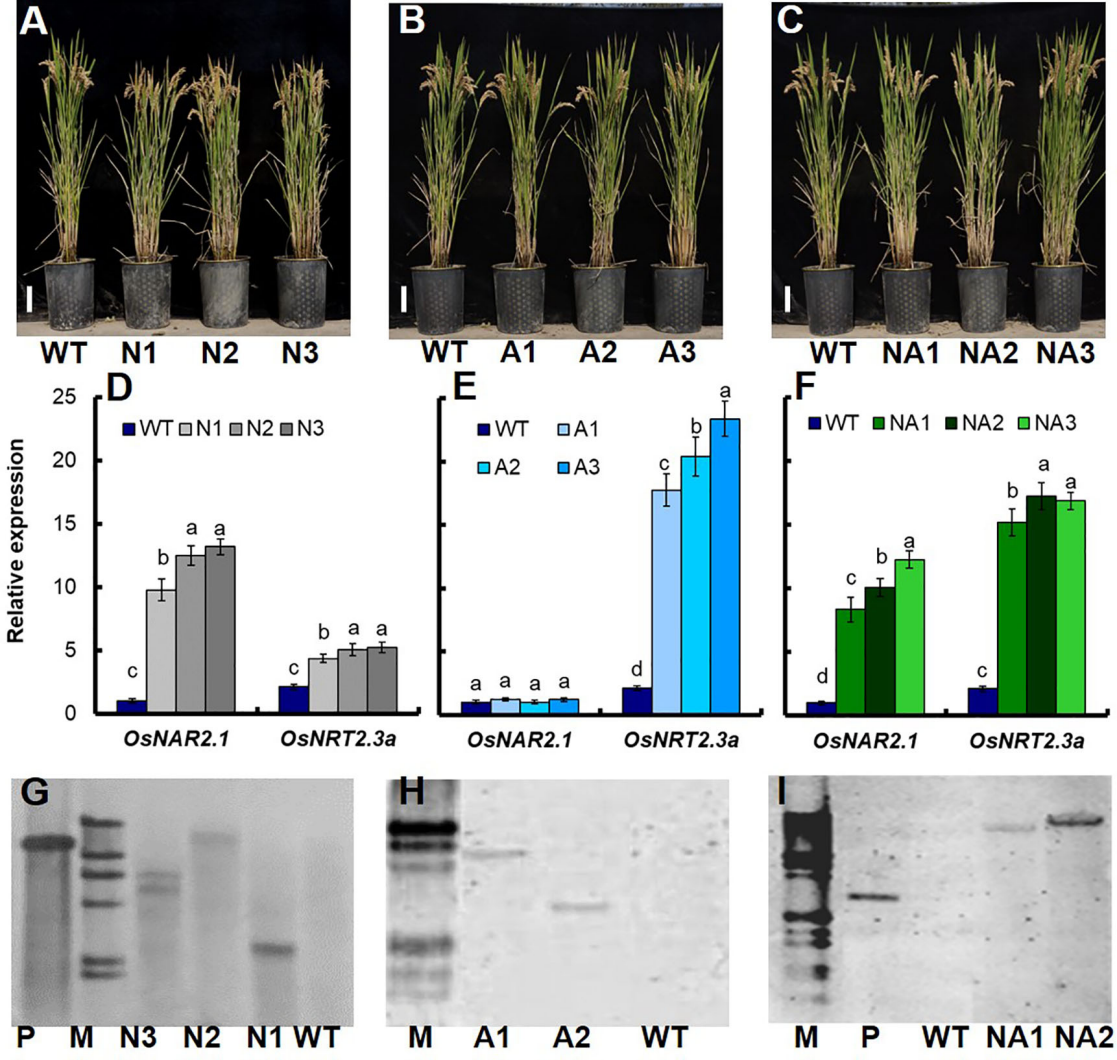
Identification of transgenic lines. Phenotype of **(A)** *p35S:OsNAR2.1* transgenic lines (N1, N2, and N3), **(B)** *p35S:OsNRT2.3a* transgenic lines (A1, A2, and A3), and **(C)** *p35S:OsNAR2.1-p35S:OsNRT2.3a* transgenic lines (NA1, NA2, and NA3). qRT-PCR analysis the expression of *OsNAR2.1* and *OsNRT2.3a* of **(D)** *p35S:OsNAR2.1* transgenic lines, **(E)** *p35S:OsNRT2.3a* transgenic lines, and **(F)** *p35S:OsNAR2.1-p35S:OsNRT2.3a* transgenic lines. RNA was extracted from culm. Error bars: SE (n = 3). The different letters indicate a significant difference between the transgenic line and the WT (P < 0.05, one-way ANOVA). Southern blot analysis the copy number of **(G)** *p35S:OsNAR2.1* transgenic line, **(H)** *p35S:OsNRT2.3a* transgenic lines, and **(I)** *p35S:OsNAR2.1-p35S:OsNRT2.3a* transgenic lines. Genomic DNA isolated from T2 generation transgenic plants was digested with the *Hind* III and *EcoR* I restriction enzymes. A G418 gene probe was used for hybridization. P, positive control; M, marker.

In this study, we further introduced the *p35S:OsNAR2.1* (**Figure S1A**), *p35S:OsNRT2.3a* (**Figure S1B**) and *p35S:OsNAR2.1-p35S:OsNRT2.3a* (**Figure S1C**) expression construction into Wuyunjing7 (WT) which is a high yield rice cultivar used in Jiangsu, China. We generated 18 lines, including 6 *p35S:OsNAR2.1* lines (N lines), 6 *p35S:OsNRT2.3a* lines (A lines), and 6 *p35S:OsNAR2.1-p35S:OsNRT2.3a* lines (NA lines). Then we conducted field experiments and analyzed the grain yield and biomass of these transgenic lines. T1–T2 generation transgenic plants and WT plants were planted in the plots with 300 kg N/ha in Nanjing, Jiangsu. Compared to WT plants, the yield and biomass for N lines of T1 plants increased by approximately 17.4% and 7.1%, respectively (**Figure S2A**). However, there was no significant difference between A lines and WT (**Figure S2B**). The yield and biomass of NA lines of T1 plants increased by approximately 30.5% and 19.5%, respectively (**Figure S2C**). Based on RNA expression data for the T1 generation (**Figures S2D–F**) and T2 generation (**Figures 1D–F**), we selected three N T2 lines named N1, N2, N3 (**Figure 1A**), three A T2 lines named A1, A2, A3 (**Figure 1B**), and three NA T2 lines named NA1, NA2, NA3 (**Figure 1C**) for further analysis. Southern blot analysis showed that N1 and N2 were two independent transgenic lines with one transgenic copy respectively (**Figure 1G**); A1 and A2 were two independent transgenic lines with one transgenic copy respectively (**Figure 1H**); NA1 and NA2 were two independent transgenic lines with one transgenic copy respectively (**Figure 1I**).

For the T3 transgenic plants were planted in the plots with 150 kg N/ha in Changxing, Zhejiang. With WT as control, the grain yield of N (N1 and N2) lines was increased by 14.5%, and agronomic nitrogen use efficiency increased by 15.1% (**Figure S3**). The grain yield and agronomic nitrogen use efficiency of NA (NA1 and NA2) lines increased by 27.1% and 31.4% compared with WT (**Figure S3**). There was no significant difference in grain yield and agronomic nitrogen use efficiency between A lines and WT (**Figure S3**).

The data of the field experiment for T4 generation which planted in the plots with 300 kg N/ha in Nanjing, Jiangsu Province were analyzed in detail. Compared with WT, the total tiller number per plant and seed setting rate increased by 26.2% and 16.3% respectively, while the panicle length, grain weight and grain number per panicle was reduced by 4.9%, 11.4%, and 11.6% respectively, and the grain yield increased by 14.1% (**Table 1**). For the NA lines, there was no significant difference in seed setting rate and tiller number between and N lines, but the grain yield increased by 24.6% (**Table 1**) compared to the control. However, there was no significant difference between all agronomic traits of A lines and WT (**Table 1**).

**Table 1:** Comparison of agronomic traits of transgenic lines.

Genotype	WT	N1	N2	A1	A2	NA1	NA2
Total tiller number per plant	20.48b	26.25a	25.80a	21.11b	20.96b	25.44a	25.92a
Panicle length (cm)	13.78a	13.05b	13.17b	13.68a	13.78a	13.80a	13.94a
Grain weight (g/panicle)	2.32a	2.02b	2.09b	2.29a	2.34a	2.42a	2.39a
Seed setting rate (%)	72.67b	84.04a	83.33a	73.96b	74.79b	84.51a	86.22a
Grain number per panicle	130.67a	114.20b	116.87b	129.53a	131.63a	133.45a	135.28a
1000-grain weight (g)	25.79a	25.93a	25.16a	26.04a	25.72a	26.24a	25.72a
Grain yield (g/plant)	26.37c	29.98b	30.19b	25.81c	26.27c	32.55a	33.19a
Grain-straw ratio	0.97c	1.17a	1.16a	0.97c	0.96c	1.06b	1.04b

Statistical analysis of data from T4 generation; n = 3 plots for each mean. The different letters indicate a significant difference between the transgenic line and the WT. (P < 0.05, one-way ANOVA).

### Dry Matter Accumulation and N Analysis in Transgenic Lines

**Figure 2:**
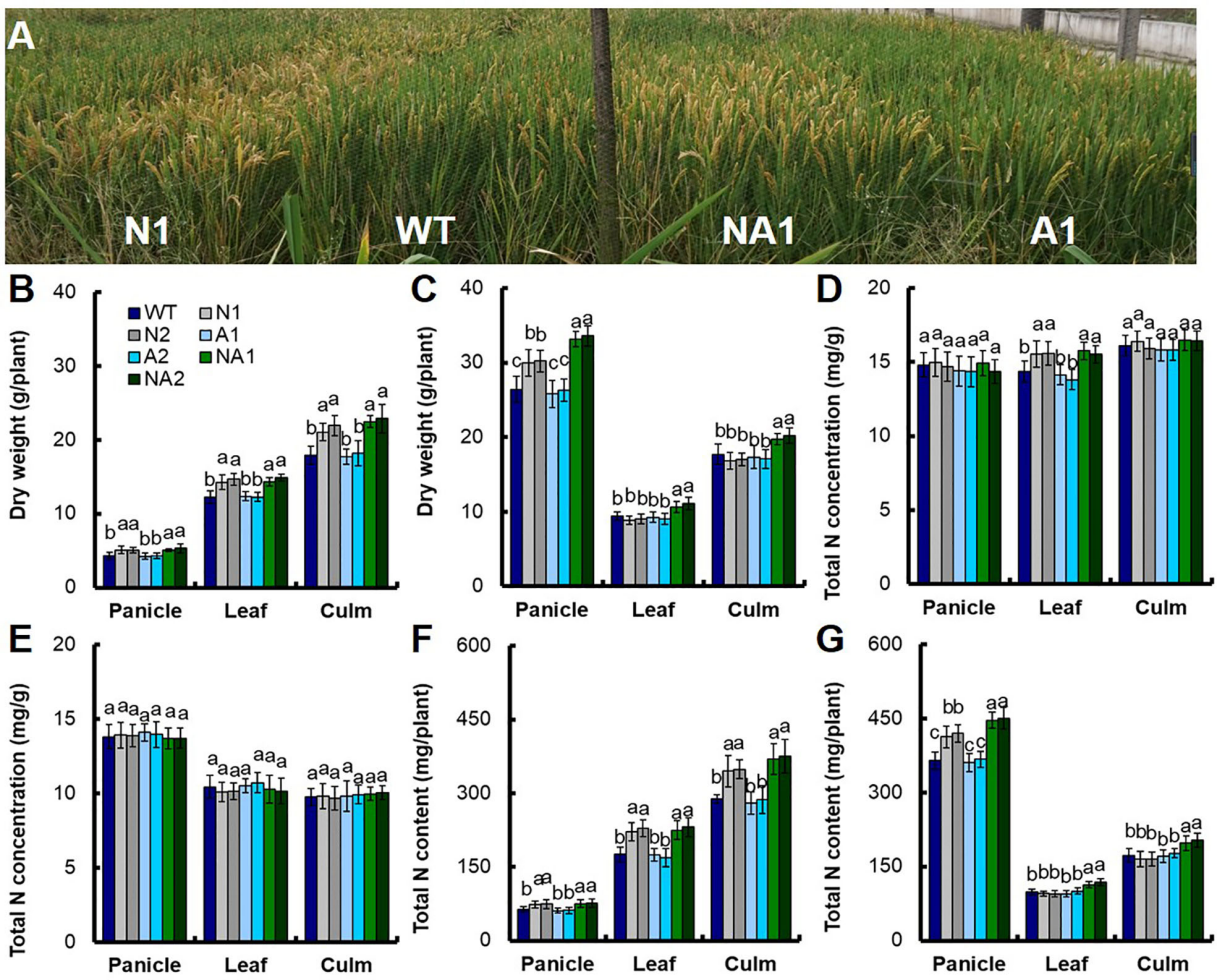
Biomass and nitrogen content in different parts of transgenic lines at the anthesis stage and maturity stage. **(A)** Photograph of wild-type (cv. Wuyunjing 7, WT) and T4 generation transgenic lines in the field experiment at Nanjing. Biomass in various parts of WT and T4 generation transgenic plants at **(B)** the anthesis stage and **(C)** maturity stage. Nitrogen concentration in different parts of transgenic lines and WT at the **(D)** anthesis stage and **(E)** maturity stage. Nitrogen content in different parts of transgenic lines and WT at the **(F)** anthesis stage, and **(G)** maturity stage. Error bars: SE (n = 3). The different letters indicate a significant difference between the transgenic line and the WT (P < 0.05, one-way ANOVA).

Anthesis stage is a particular time from vegetative growth to reproductive growth of rice, and dry matter and nitrogen accumulation during anthesis stage have positive influence on the formation of final yield of rice (Good et al., 2004). We sampled shoot tissues at the anthesis stage and at the mature stage separately to determine the biomass and the total N content. Analysis using WT as control, we found that at the anthesis stage, the biomass of panicles, leaves and culms in N lines increased by 18.7%, 18.3%, and 20.0% respectively; the biomass of panicles, leaves and culms in A lines had no significant difference; the biomass of panicles, leaves and culms in NA lines increased by 21.1%, 19.3%, and 26.6% (**Figure 2B**). At the mature stage, the biomass of panicles in N lines increased by about 14.0%, but the biomass of leaves and culms for N was not significant. There was no significant difference between the biomass of panicles, leaves and culms between A lines and WT. For the NA lines, the biomass of panicles, leaves and culms was increased by 26.4%, 15.5%, and 12.8% respectively compared with WT (**Figure 2C**).

At anthesis stage, there was no significant difference of the total nitrogen concentration in leaves between N lines and NA lines, but increased by about 8.8% compared with WT. For the total nitrogen concentration in panicles and culms, there was no significant difference between N lines, NA lines and WT. There was no significant difference in total nitrogen concentration between different parts of A lines and WT (**Figure 2D**). At maturity stage, there was no significant difference in total nitrogen concentration between N, A and NA lines and WT (**Figure 2E**).

The variation of biomass and total nitrogen concentration eventually led to the difference of total nitrogen content per plant. At anthesis stage, there was no significant difference in total nitrogen concentration in panicles, leaves and culms between N and NA lines, but increased by about 18.3%, 29.2%, and 24.7% compared to WT (**Figure 2F**). At maturity stage, the total nitrogen content in panicles of N lines increased by about 14.2%, there was no significant difference in the total nitrogen content in leaves and culms between N lines and WT. The total nitrogen content in panicles, leaves and culms of NA lines increased by about 23.0%, 17.7%, and 15.7% respectively, however, there was no significant difference in total nitrogen content between A lines and WT in different plant parts (**Figure 2G**).

Dry matter and N translocation in rice plants were investigated by determining dry matter at anthesis stage (DMA), dry matter at maturity (DMM), grain yield (GY), total N accumulation at anthesis stage (TNAA), total N accumulation at maturity stage (TNAM) and grain nitrogen content at maturity stage (GNAM) respectively. Compared with WT, the DMA, DMM, GY, TNAA, TNAM, and GNAM of N lines increased by 19.0%, 6.2%, 12.1%, 22.5%, 6.8%, and 14.9% respectively, NA lines increased by about 23.3%, 18.6%, 22.6%, 28.3%, 19.5%, and 21.9%, respectively. There were no significant differences in DMA, DMM, GY, TNAA, TNAM, and GNAM between A lines and WT (**Table 2**).

**Table 2:** Biomass and nitrogen content of transgenic lines.

	WT	N1	N2	A1	A2	NA1	NA2
DMA (kg/m2)	0.86b	1.01a	1.04a	0.86b	0.87b	1.04a	1.08a
DMM (kg/m2)	1.33c	1.41b	1.42b	1.31c	1.34c	1.56a	1.60a
GY (kg/m2)	0.66c	0.73b	0.74b	0.64c	0.65c	0.80a	0.81a
TNAA (g/m2)	13.17b	15.99a	16.28a	12.86b	12.94b	16.74a	17.06a
TNAM (g/m2)	15.87c	16.93b	16.97b	15.62c	16.01c	18.74a	19.19a
GNAM (g/m2)	9.11c	10.43b	10.49b	9.01c	9.17c	11.03a	11.17a

Dry matter at anthesis (DMA), dry matter at maturity (DMM), grain yield (GY), total nitrogen accumulation at anthesis (TNAA), total nitrogen accumulation at maturity (TNAM), and grain nitrogen accumulation at maturity (GNAM). Statistical analysis of data from T4 generation; n = 3 plots for each mean. The different letters indicate a significant difference between the transgenic line and the WT. (P < 0.05, one-way ANOVA).

### Nitrogen Use Efficiency Estimation of the Transgenic Lines

We also detected dry matter translocation (DMT), DMT efficiency (DMTE), the contribution of pre-anthesis assimilates to grain yield (CPAGY), nitrogen translocation (NT), NT efficiency (NTE), and the contribution of pre-anthesis N to grain N accumulation (CPNGN), based on a method described by Chen et al. (2016). Compared with WT, DMT, DMTE, CPAGY, NT, NTE, and CPNGN of N lines increased by 106.5%, 73.5%, 81.1%, 50.6%, 22.9%, and 31.1%, respectively, NA lines increased by 59.1%, 28.9%, 29.7%, 40.8%, 9.8%, and 15.7%, respectively. There were no significant differences in DMT, DMTE, CPAY, NT, NTE, and CPNGN between A lines and WT (**Table 3**).

**Table 3:** Dry matter transport efficiency, N transport efficiency, and N-use efficiency of transgenic lines.

	WT	N1	N2	A1	A2	NA1	NA2
DMT (g/m^2^)	182.32c	363.04a	389.77a	192.34c	203.39c	284.88b	295.08b
DMTE (%)	21.20c	36.13a	37.44a	22.45c	19.86c	27.28b	27.40b
CPAGY (%)	27.62c	48.43a	51.63a	29.81c	27.34c	35.44b	36.21b
HI (%)	49.35b	53.88a	53.68a	49.27b	48.83b	51.21ab	51.03ab
NT (g/m^2^)	6.40c	9.49a	9.79a	6.25c	6.02c	8.99b	9.04b
NTE (%)	48.61c	59.33a	60.17a	48.59c	46.53c	53.74b	53.02b
CPNGN (%)	70.32c	91.00a	93.43a	69.39c	65.66c	81.80b	80.95b
NHI (%)	57.36b	61.59a	61.79a	57.16b	56.99b	58.68ab	58.22ab
PNUE (g/g)	51.77a	53.59a	54.37a	51.85a	50.16a	53.47a	53.34a

Dry matter translocation (DMT), dry matter translocation efficiency (DMTE), the contribution of pre-anthesis assimilates to grain yield (CPAGY), harvest index (HI), nitrogen translocation (NT), nitrogen translocation efficiency (NTE), contribution of pre-anthesis nitrogen to grain nitrogen accumulation (CPNGN), nitrogen harvest index (NHI) and physiological nitrogen use efficiency (PNUE). Statistical analysis of data from T4 generation; n = 3 plots for each mean. The different letters indicate a significant difference between the transgenic line and the WT. (P < 0.05, one-way ANOVA).

**Figure 3:**
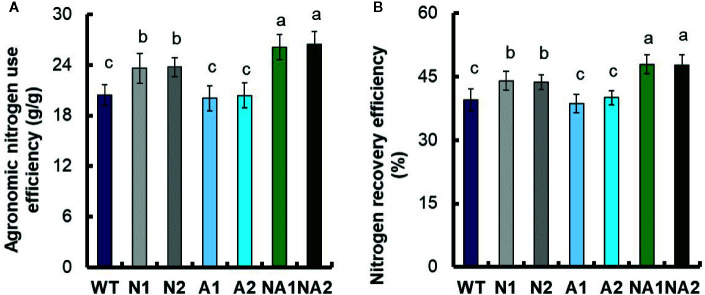
Comparison of nitrogen use efficiency between the WT and transgenic lines. **(A)** Agronomic nitrogen use efficiency (ANUE), **(B)** nitrogen recovery efficiency (NRE). Error bars: SE (n = 3). The different letters indicate a significant difference between the transgenic line and the WT (P < 0.05, one-way ANOVA).

Based on a method described by Chen et al. (2016), we calculated the physiological nitrogen use efficiency (PNUE), nitrogen harvest index (NHI), harvest index (HI), agronomic nitrogen use efficiency (ANUE) and nitrogen recovery efficiency (NRE). Compared with WT, the PNUE of N lines did not change significantly, NHI and HI increased by about 7.6% and 9.0% respectively; the PNUE, NHI, and HI of A and NA lines had no significant difference compared with WT (**Table 3**). But the ANUE and NRE of N lines increased by 15.8% and 11.1% respectively; the NA lines increased by 28.6% and 21.2% respectively, while the ANUE and NRE of A lines did not change compared with WT (**Figures 3A, B** **)**.

### The Expression of *OsNAR2.1* and *OsNRT2s* in Transgenic Lines in Different Growth Stages of WT and Transgenic Lines

**Figure 4:**
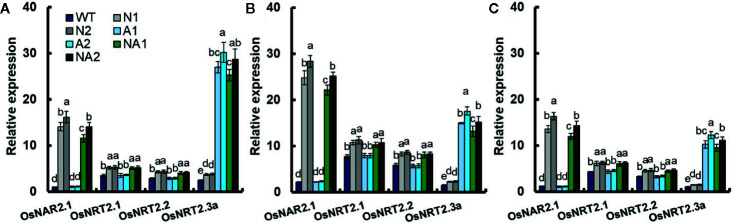
Expression of *OsNAR2.1* and *OsNAR2s* in transgenic lines and WT during the experimental growth period. Samples were collected **(A)** 30 days (the vegetative growth period), **(B)** 60 days (the anthesis stage) and **(C)** 75 days (the grain filling stage) after seedlings were transplanted to the field. RNA was extracted from culms. Error bars: SE (n = 3). The different letters indicate a significant difference between the transgenic line and the WT (P < 0.05, one-way ANOVA).

Chen et al. (2016, 2017) reported that the expression of *OsNAR2.1* and *OsNRT2.1* in culms significantly affected rice yield and nitrogen use efficiency. In this study, we analyzed the expression of *OsNAR2.1* and *OsNRT2s* in culms of WT and transgenic lines at 30 days (the vegetative growth period), 60 days (the anthesis stage), and 75 days (the grain filling stage) separately. During these three periods, the expressions of *OsNAR2.1* and *OsNRT2.3a* of N (N1, N2, and N3) or NA (N1, NA2, and N3) lines were significantly higher compared with WT. But there was no significant difference of the expressions of *OsNRT2.1*and *OsNRT2.2* between N and NA lines (**Figure 4**). Compared with WT, the expression of *OsNAR2.1* increased by about 14 times in N lines, while *OsNRT2.3a* increased by about 51% in those lines. The expression of *OsNAR2.1* and *OsNRT2.3a* in NA lines increased about 12-fold and 10-fold respectively (**Figure 4**). Compared with WT, the expression of *OsNRT2.3a* in A (A1, A2, and A3) lines increased about 10 times, while the expression of *OsNAR2.1, OsNRT2.1*, and *OsNRT2.2* did not change significantly (**Figure 4**).

### Agronomic Nitrogen Use Efficiency Estimation for Co-Overexpression of *OsNAR2.1* and *OsNRT2.3a* in the cv. Nipponbare Rice

**Figure 5:**
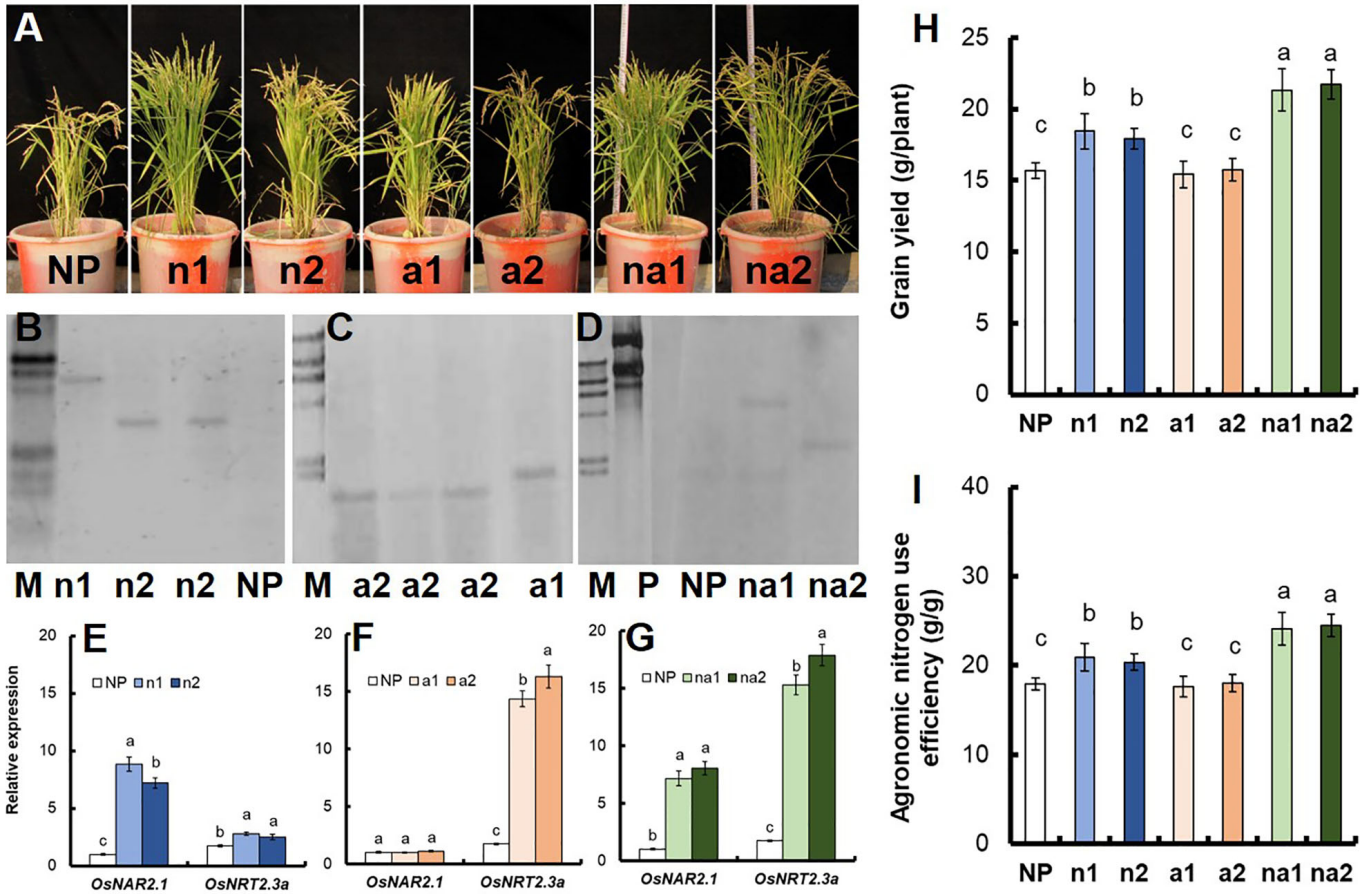
Grain yield and agronomic nitrogen use efficiency of Nipponbare transgenic lines. **(A)** Phenotype of wild-type (cv. Nipponbare, NP), *p35S:OsNAR2.1* transgenic lines (n1 and n2), *p35S:OsNRT2.3a* transgenic lines (a1 and a2), and *p35S:OsNAR2.1-p35S:OsNRT2.3a* transgenic lines (na1, na2). Southern blot analysis the copy number of **(B)** *p35S:OsNAR2.1* transgenic lines, **(C)** *p35S:OsNRT2.3a* transgenic lines, and **(D)** *p35S:OsNAR2.1-p35S:OsNRT2.3a* transgenic lines. Genomic DNA isolated from T2 generation transgenic plants was digested with the *Hind* III and *EcoR* I restriction enzymes. A G418 gene probe was used for hybridization. P, positive control; M, marker. qRT-PCR analysis the expression of *OsNAR2.1* and *OsNRT2.3a* of **(E)** *p35S:OsNAR2.1* transgenic lines, **(F)** *p35S:OsNRT2.3a* transgenic lines, and **(G)** *p35S:OsNAR2.1-p35S:OsNRT2.3a* transgenic lines. RNA was extracted from culm. Comparison of **(H)** grain yield and **(I)** agronomic nitrogen use efficiency between the NP and transgenic lines. Error bars: SE (n = 3). The different letters indicate a significant difference between the transgenic line and the WT (P < 0.05, one-way ANOVA).

We also introduced the *p35S:OsNAR2.1* (**Figure S1A**), *p35S:OsNRT2.3a* (**Figure S1B**), and *p35S:OsNAR2.1-p35S:OsNRT2.3a* (**Figure S1C**) expression construct into Nipponbare cultivar. We generated 6 lines, including 2 *p35S:OsNAR2.1* lines (n lines), 2 *p35S:OsNRT2.3a* lines (a lines), and 2 *p35S:OsNAR2.1-p35S:OsNRT2.3a* lines (na lines) (**Figure 5C**). Southern blot analysis showed that n1 and n2 were two independent transgenic lines with one transgenic copy respectively (**Figure 5B**); a1 and a2 were two independent transgenic lines with one transgenic copy respectively (**Figure 5C**); na1 and na2 were two independent transgenic lines with one transgenic copy respectively (**Figure 5D**). Based on Southern blot analysis and gene expression analysis (**Figures 5E–G**) of T2 plants, they are independent lines. Compared with NP (Nipponbare), the grain yields of n lines and na lines increased by 16.0% and 37.3% (**Figure 5H**), and the agronomic nitrogen use efficiency increased by 14.9% and 35.2% (**Figure 5I**), respectively.

### The Plant Seedling Growth and Total Nitrogen Content Evaluation in Transgenic Lines

**Figure 6:**
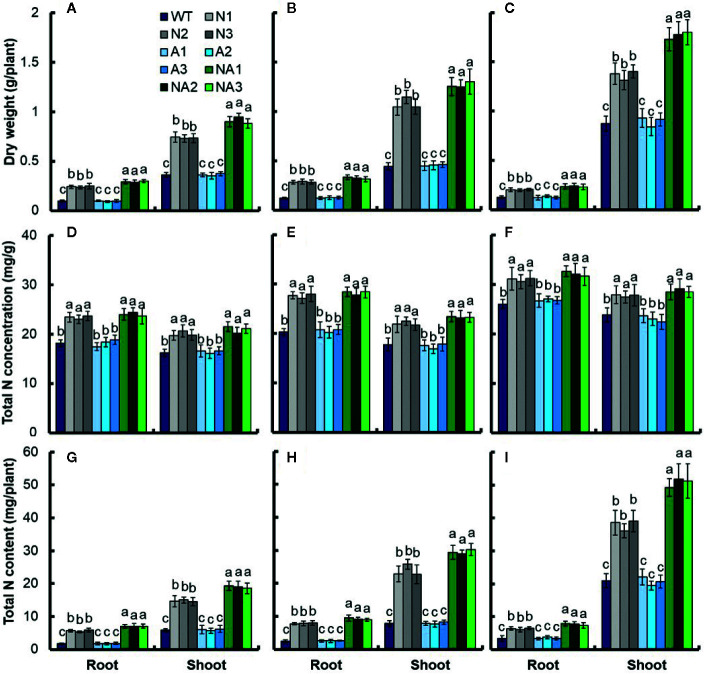
Total nitrogen content of transgenic plants at different nitrogen supply levels. WT and transgenic rice seedlings in the solution containing 1 mM NH_4_^+^ of IRRI for 2 weeks, and in different forms of nitrogen for 3 additional weeks. Dry weight of seedlings treated with **(A)** 0.2 mM NO_3_^–^, **(B)** 2.5 mM NO_3_^–^, and **(C)** 1.25 mM NH_4_NO_3_; Total nitrogen concentration of seedlings treated with **(D)** 0.2 mM NO_3_^–^, **(E)** 2.5 mM NO_3_^–^, and **(F)** 1.25 mM NH_4_NO_3_; Total N content of seedlings grown with **(G)** 0.2 mM NO_3_^–^, **(H)** 2.5 mM NO_3_^–^ and **(I)** 1.25 mM NH_4_NO_3_. Error bars: SE (n = 4). The different letters indicate a significant difference between the transgenic line and the WT (P < 0.05, one-way ANOVA).

We further analyzed the growth and nitrogen uptake of transgenic lines at seedling stage. WT and transgenic rice seedlings were grown in IRRI solution containing 1 mM NH_4_^+^ for 2 weeks and then transferred into 0.2 mM NO_3_^–^, 2.5 mM NO_3_^–^, or 1.25 mM NH_4_NO_3_ for 3 additional weeks (**Figure S4**). Compared with WT, under 0.2 mM NO_3_^–^ treatment, the biomass of root and shoot in N lines increased 151.2% and 102.7% respectively, it increased 204.0% and 150.7% respectively in NA lines (**Figure 6A**). Under 2.5 mM NO_3_^–^ treatment, the biomass of root and shoot in N lines increased 136.8% and 142.8% respectively, it increased 170.1% and 185.7% respectively in NA lines (**Figure 6B**). Under 1.25 mM NH_4_NO_3_ treatment, the biomass of root shoot in N lines increased 58.9% and 56.2% respectively, it increased 85.5% and 102.1% respectively in NA lines (**Figure 6C**).

Moreover, we measured the total nitrogen concentration, and found that there was no significant difference in total nitrogen concentration between N lines and NA lines under different treatments. Compared with WT, the total nitrogen concentration in root and shoot of N lines or NA lines increased by 30.5% and 26.6% respectively under 0.2 mM NO_3_^–^ treatment (**Figure 6D**), increased by 37.4% and 27.4% respectively under 2.5 mM NO_3_^–^ treatment (**Figure 6E**), increased by 21.1% and 18.0% respectively under 1.25 mM NH_4_NO_3_ treatment (**Figure 6F**).

The total nitrogen content per plant was also calculated. After 0.2 mM NO_3_^–^ treatment, the total nitrogen content of the root and shoot in N lines increased 223.5% and 151.2% respectively, it increased 302.0% and 224.0% respectively in NA lines (**Figure 6G**). After 2.5 mM NO_3_^–^ treatment, the total nitrogen content increased 221.7% and 201.2% respectively in N lines, it increased 275.0% and 273.9% respectively NA lines (**Figure 6H**); under 1.25 mM NH_4_NO_3_ treatment, the total nitrogen content increased 89.1% and 81.3% respectively in N lines, it increased 128.7% and 142.7% respectively in NA lines (**Figure 6I**).

We further analyzed the expression of *OsNAR2s* and *OsNRT2s* in transgenic lines treated with 0.2 mM NO_3_^–^ and 1.25 mM NH_4_NO_3_. The expression patterns of the transgenic lines were similar under the treatment of 0.2 mM NO_3_^–^ and 1.25 mM NH_4_NO_3_. In the N lines, the expression of *OsNAR2.1* increased by about 12.4 times compared with WT, and the expression of *OsNRT2.1, OsNRT2.2*, and *OsNRT2.3a* increased by 55%-98% (**Figures S5A, D** **)**. In the A lines, the expression of *OsNRT2.3a* in increased about 11 times, but the expression of *OsNAR2.1, OsNRT2.2*, and *OsNRT2.2* did not change significantly (**Figures S5B, E** **)**. However, for the NA lines, the expression of *OsNAR2.1* increased by 13.8 times, the expression of *OsNRT2.3a* increased by 10.1 times, and the expression of *OsNRT2.1* and *OsNRT2.2* increased by 53%-120% (**Figures S5C, F** **)**. The expression of *OsNAR2.2*, *OsNRT2.3b* and *OsNRT2.4* did not change significantly in N, A, and NA lines (**Figure S6**).

### ^15^N Influx Rates and ^15^N Distribution Ratio Influx Determination

**Figure 7:**
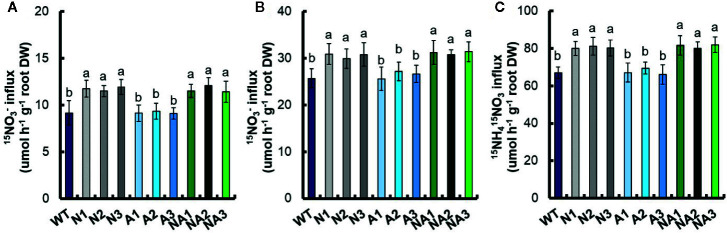
^15^N influx rates of transgenic lines. WT and transgenic seedlings were grown in 1 mM NH_4_^+^ for 3 weeks and nitrogen starved for 1 week. ^15^N influx rates were then measured at **(A)** 0.2 mM ^15^NO_3_^–^, **(B)** 2.5 mM ^15^NO_3_^–^, and **(C)** 1.25 mM ^15^NH_4_^15^NO_3_ during 5 min. DW, dry weight. Error bars: SE (n = 4). The different letters indicate a significant difference between the transgenic line and the WT (P < 0.05, one-way ANOVA).

We analyzed short-term ^15^N uptake in same-size seedlings of the transgenic lines and WT which exposed to 0.2 mM ^15^NO_3_^–^, 2.5 mM ^15^NO_3_^–^, or 1.25 mM ^15^NH_4_^15^NO_3_ for 5 min. There was no significant difference of ^15^N influx rate between N lines and A lines. Compared with WT, the influx rate of ^15^NO_3_^–^ increased 27.6% and 20.1% in response to 0.2 mM ^15^NO_3_^–^ and 2.5 mM ^15^NO_3_^–^, respectively; the influx rate of ^15^NH_4_^15^NO_3_ increased 20.6% responded to 1.25 mM ^15^NH_4_^15^NO_3_ (**Figures 7A–C**). The influx rate of ^15^N did not change compared with that of WT in the A lines (**Figures 7A–C**).

**Figure 8:**
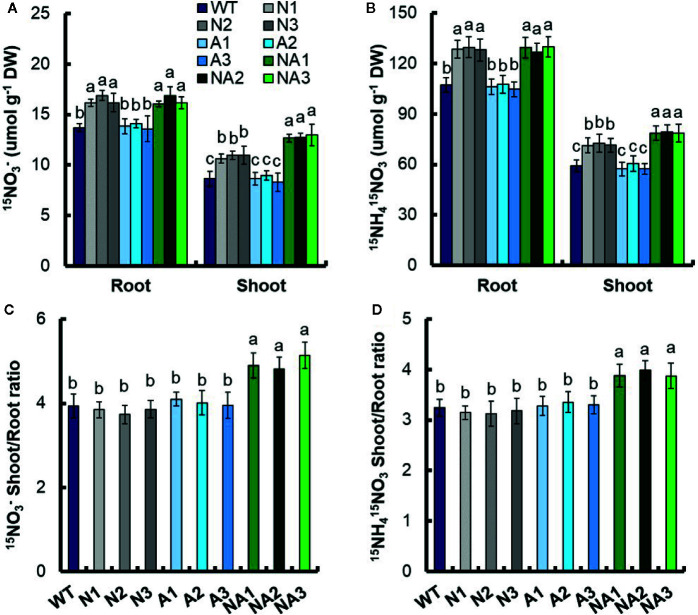
^15^N concentration and ^15^N distribution ratio in WT and transgenic lines. WT and transgenic seedlings were grown in 1 mM NH_4_^+^ for 3 weeks and nitrogen starved for 1 week. ^15^N concentration in the roots and shoots of the WT and transgenic lines under the nutrient solution containing **(A)** 0.2 mM ^15^NO_3_^–^ or **(B)** 1.25 mM ^15^NH_4_^15^NO_3_ for 12 h. The shoot-to-root ratio of the total ^15^N content at **(C)** 0.2 mM NH_4_^+^ or **(D)** 1.25 mM ^15^NH_4_^15^NO_3_. DW, dry weight. Error bars: SE (n = 4). The different letters indicate a significant difference between the transgenic line and the WT (P < 0.05, one-way ANOVA).

We also detected ^15^N distribution ratio experiment in root. WT and transgenic seedlings were grown in 1 mM NH_4_^+^ for 3 weeks and treated in nitrogen starvation condition for 1 week. ^15^N concentration experiment was conducted under the nutrient solution containing 0.2 mM ^15^NO_3_^–^ or 1.25 mM ^15^NH_4_^15^NO_3_ for 12 h. Under 0.2mM ^15^NO_3_^–^ treatment, there was no significant difference of the ^15^N concentration in roots between N and NA lines, but the ^15^N concentration increased by 19.7% compared with WT. ^15^N concentration in roots of N and NA lines increased by 25.5% and 48.1%, respectively compared with WT (**Figure 8A**). Under 1.25 mM ^15^NH_4_^15^NO_3_ treatment, there was no significant difference in ^15^N concentration in roots between N and NA lines, but it increased by 20.0% compared with WT. ^15^N concentration in roots of N and NA lines increased by 21.2% and 33.2%, respectively (**Figure 8B**). Under the treatment of 0.2 mM ^15^NO_3_^–^ and 1.25 mM ^15^NH_4_^15^NO_3_, there was no significant difference in ^15^N concentration in roots and shoots between A lines and WT (**Figures 8A, B** **)**. Finally, compared with WT, the ^15^N of shoot to root ratio in the NA lines increased by 25.9% and 20.7% respectively under 0.2 mM ^15^NO_3_^–^ and 1.25 mM ^15^NH_4_^15^NO_3_ treatments (**Figures 8C, D** **)**. There was no significant difference of the ^15^N in shoot to root ratio between WT, N lines and A lines under 0.2 mM ^15^NO_3_^–^ or 1.25 mM ^15^NH_4_^15^NO_3_ (**Figures 8C, D** **)**.

## Discussion

Increasing NO_3_^–^ uptake is an effective way to improve yield and nitrogen use efficiency in rice. Fang et al. (2013) found that overexpression of nitrate transporter *OsPTR9* could promote lateral root formation, increase grain yield of rice. Overexpression of *OsNRT1.1B*, a low affinity NO_3_^–^ transporter protein gene, increased rice nitrogen use efficiency by about 30% (Hu et al., 2015). Overexpression of high affinity nitrate transporter gene *OsNRT2.3b* increased the buffer capacity of cell pH and significantly increased grain yield and nitrogen use efficiency of rice (Fan et al., 2016). Overexpression of nitrate transporter gene *OsNRT1.1A* in rice greatly improved nitrogen utilization and grain yield, and maturation time was also significantly shortened (Wang et al., 2018). Gao et al. (2019) reported that overexpression of *OsNR2* increased the activity of nitrate reductase and the uptake of nitrate by rice, thus increasing the yield and nitrogen use efficiency of rice. Overexpression of *OsNAR2.1* or *OsNRT2.1* by the native promoter of *OsNAR2.1* can improve grain yield and nitrogen use efficiency of rice (Chen et al., 2016; Chen et al., 2017; Luo et al., 2018; Chen et al., 2019). *OsNAC42* as a transcription factor enhances nitrate uptake in rice by regulating nitrate transporter gene *OsNPF6.1*, thereby enhancing the regulation of rice nitrogen use efficiency (Tang et al., 2019). In this study, we investigated the effect of co-overexpression of *OsNAR2.1* and *OsNRT2.3a* on NO_3_^–^ uptake, yield and nitrogen use efficiency uptake by rice.

OsNAR2.1 act as a partner protein with OsNRT2s for transport of NO_3_^–^ in rice (Yan et al., 2011; Tang et al., 2012; Liu et al., 2014). Chen et al. (2017) found that *pOsNAR2.1:OsNAR2.1* increased the expression of *OsNAR2.1* by 3‑5 times in rice, and in this study the expression of *OsNAR2.1* in *p35S:OsNAR2.1* increased by more than 10 times (**Figure 1D**, **Figure 4**, **Figures S6A, D** **)**. This because different promoters lead to different expression patterns of *OsNAR2.1*, which leads to inconsistent growth phenotypes of transgenic rice. Compared with WT, the DMA, DMM, GY, TNAA, TNAM, and GNAM of *p35S:OsNAR2.1* lines increased by 19.0%, 6.2%, 12.1%, 22.5%, 6.8%, and 14.9% respectively (**Table 2**), of *pOsNAR2.1:OsNAR2.1* lines increased by 25%, 25%, 24%, 33%, 34%, and 35% (Chen et al., 2017). This result also accords with our previous conclusion that controlling the expression ratio of *OsNAR2.1* and *OsNRT2.1* in rice in an appropriate proportion can improve more grain yield and nitrogen use efficiency in rice (Chen et al., 2016; Chen et al., 2017).

Compared with WT, the dry matter and total nitrogen accumulation of NA lines increased significantly at anthesis and maturity (**Figure 2**, **Table 2**), the DMTE, NTE, CPAGY, and CPNGN increased by 28.9%, 9.8%, 29.7%, and 15.7% respectively (**Table 3**). During rice filling, 70%‑90% of nitrogen was transported from vegetative organs to panicles (Yoneyama et al., 2016). The accumulation of nitrogen and biomass in early stage has a great influence on rice yield. Compared with WT, the GY, ANUE and NRE of NA lines increased by 22.6%, 28.6% and 21.2% respectively (**Table 3**, **Figure 3**). Compared with WT, the GY, ANUE, and NRE of N lines increased by 12.1%, 15.8%, and 11.1% respectively (**Table 3**, **Figure 3**), which was significantly lower than that of NA lines. Similarly, co-overexpression of *OsNAR2.1* and *OsNRT2.3a* increased agronomic nitrogen use efficiency of cv. Nipponbare rice. Compared with Nipponbare rice, the grain yields of *p35S:OsNAR2.1* and *p35S:NAR2.1-p35S:NRT2.3a* lines increased by 16.0% and 37.3% (**Figure 5H**), and the agronomic nitrogen use efficiency increased by 14.9% and 35.2% (**Figure 5I**), respectively. This means the co-overexpression of *OsNAR2.1* and *OsNRT2.3a* transgenic plants have functions in different rice varieties and it can provide a physiological basis for rice breeding.

Kronzucker et al. (2000) used ^13^N to show that the presence of NO_3_^–^ promotes NH_4_^+^ uptake, accumulation, and metabolism in rice. Duan et al. (2006) found that increasing NO_3_^–^ uptake promotes dry weight and NO_3_^–^ accumulation and assimilation of NH_4_^+^ and NO_3_^–^ by ‘Nanguang’, which is an N-efficient rice cultivar, during the entire growth period. Li et al. (2006) showed that supplying NH_4_^+^ and NO_3_^–^ enhances *OsAMT1;3*, *OsAMT1;2*, and *OsAMT1;1* expression compared with supplying only NH_4_^+^ or NO_3_^–^, thereby enhancing NH_4_^+^ uptake by rice. The influx rates of ^15^NH_4_^+^ and ^15^NO_3_^–^ in *pOsNAR2.1:OsNAR2.1* transgenic lines increased by 21 and 22% in 1.25 mM ^15^NH_4_NO_3_ and 1.25 mM NH_4_^15^NO_3_, respectively (Chen et al., 2017). Further studies showed that when 0.2 mM NO_3_^–^, 2.5 mM NO_3_^–^ or 1.25 mM NH_4_NO_3_ were provided as the sole nitrogen source, the dry weight and total nitrogen content of NA lines were higher than that of N lines (**Figure 6**). In the 5-min ^15^N absorption experiment, we found that there was no significant difference in the influx rate of ^15^N between N and NA strains at 0.2 mM ^15^NO_3_^–^, 2.5 mM ^15^NO_3_^–^, or 1.25 mM ^15^NH_4_^15^NO_3_ (**Figures 7A–C**). Compared with N lines, the ^15^N shoot to root ratio of NA lines increased by 25.9% and 20.7% respectively under 0.2 mM ^15^NO_3_^–^ and 1.25 mM ^15^NH_4_^15^NO_3_ treatments for 12 h (**Figures 8C, D** **)**. *OsNRT2.1* and *OsNRT2.2* are responsible for NO_3_^–^ uptake at the roots, while *OsNRT2.3a* is responsible for NO_3_^–^ transport from roots to shoots (Feng et al., 2011; Yan et al., 2011; Tang et al., 2012; Chen et al., 2016). Overexpression of *OsNAR2.1* can increase the NO_3_^–^ and NH_4_NO_3_ uptake from roots (**Figures 8A, B** **)**, but it cannot increase the transport ratio from roots to shoots (**Figures 8C, D** **)**, this because more *OsNRT2.3a* needs to be expressed in order to increase the roots-to-shoots transport ratio. In this study, we also found that overexpression of *OsNRT2.3a* alone did not change rice growth. There was no significant difference in NO_3_^–^ uptake, agronomic traits, biomass and nitrogen accumulation between A lines and WT (**Tables 1** and **2**, **Figure 2**, **Figure S3**). The reason may be that the expression of *OsNRT2.3a* alone did not affect the expression of *OsNAR2.1* (**Figures 1** and **4**, **Figure S5**).

In conclusion, co-overexpression of *OsNAR2.1* and *OsNRT2.3a* could increase the uptake as well as the transport rate of NO_3_^–^ and NH_4_NO_3_ from roots to shoots, eventually leading to increasing the total nitrogen content and biomass of rice seedlings under 0.2 mM NO_3_^–^, 2.5 mM NO_3_^–^, and 1.25 mM NH_4_NO_3_ conditions. Field experiments also showed that co-overexpression of *OsNAR2.1* and *OsNRT2.3a* could increase rice biomass and total nitrogen accumulation, as well as improve rice dry matter transport efficiency and nitrogen transport efficiency, inducing improving rice yield, agronomic nitrogen use efficiency and nitrogen recovery efficiency in rice. Through co-overexpression of *OsNAR2.1* and *OsNRT2.3a* have the same advantages in different rice varieties, it may also function in different crops. This approach provides an effective way to improve grain yield and nitrogen use efficiency in plant.

## Data Availability Statement

The raw data supporting the conclusions of this article will be made available by the authors, without undue reservation.

## Author Contributions

Conceived and designed the experiments: JC, XL, XiaoroF, and GX. Performed the experiments: JC, XL, SL, XiaoruF, LZ, and MS. Analyzed the data: JC, XL, and XiaoroF. Wrote and revised the paper: JC, XL, XiaoroF, and GX.

## Funding

This work is supported by the Transgenic Project (Grant 2016ZX08001003-008), the Fundamental Research Funds for the Central Universities (KYZ202006), Innovative Research Team Development Plan of Ministry of Education of China (IRT_17R56; KYT201802), Jiangsu Science and Technology Development Program (Grant BE2019375-1), and Dapeng District Industry Development Special Funds (KY20180218).

## Conflict of Interest

The authors declare that the research was conducted in the absence of any commercial or financial relationships that could be construed as a potential conflict of interest.
