# Supplementary material for: Co-Overexpression of OsNAR2.1 and OsNRT2.3a Increased Agronomic Nitrogen Use Efficiency in Transgenic Rice Plants
Source: Front Plant Sci. 2020 Aug 12;11:1245. doi: 10.3389/fpls.2020.01245 (PMC7434940; doi:10.3389/fpls.2020.01245)
Supplement: Supplementary file 1 [file Presentation_1.ppt]

## Slide 1
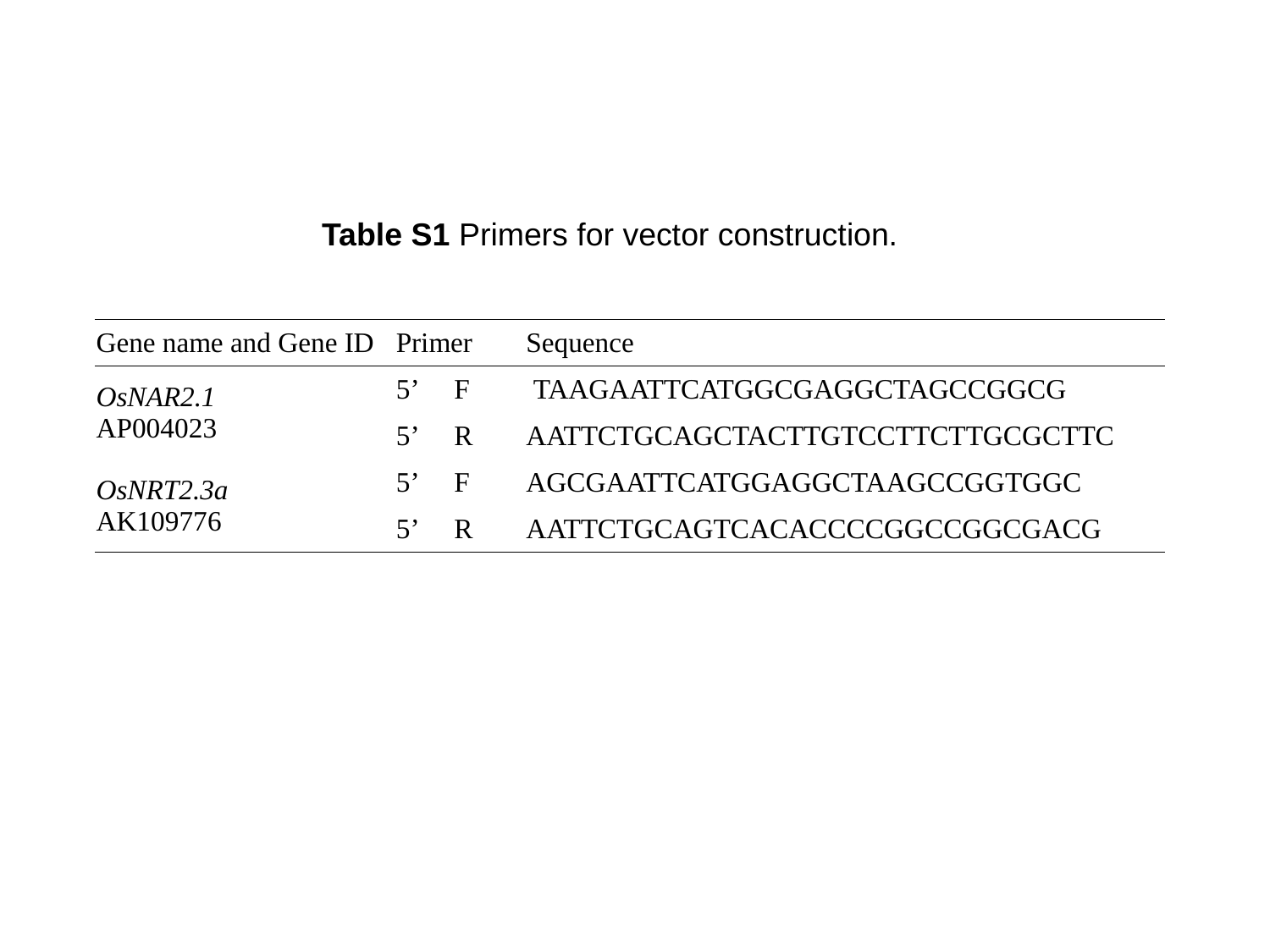

Table S1 Primers for vector construction.
| Gene name and Gene ID | Primer | Sequence |
| --- | --- | --- |
| OsNAR2.1 AP004023 | 5’（F） | TAAGAATTCATGGCGAGGCTAGCCGGCG |
| | 5’（R） | AATTCTGCAGCTACTTGTCCTTCTTGCGCTTC |
| OsNRT2.3a AK109776 | 5’（F） | AGCGAATTCATGGAGGCTAAGCCGGTGGC |
| | 5’（R） | AATTCTGCAGTCACACCCCGGCCGGCGACG |

## Slide 2
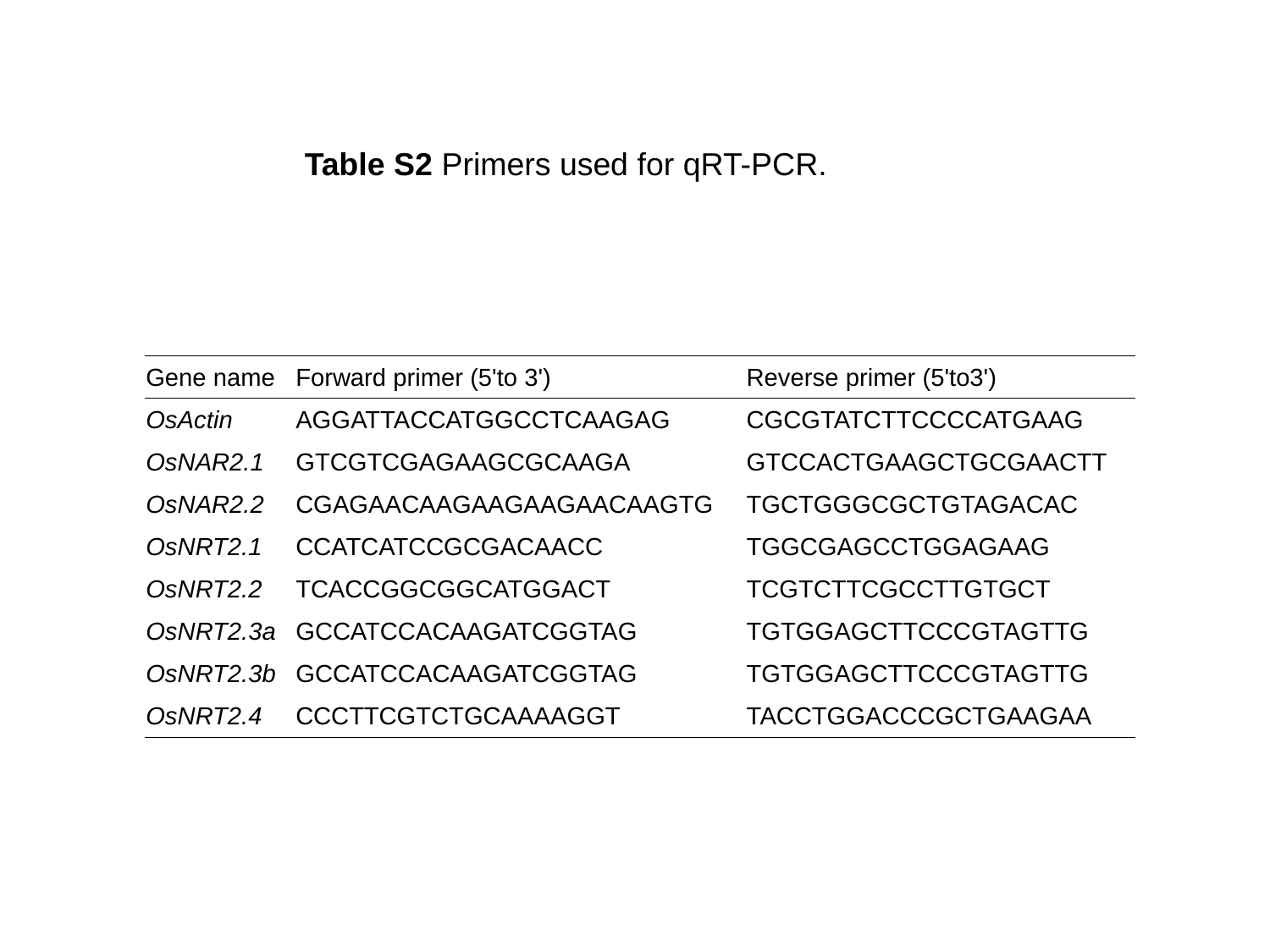

Table S2 Primers used for qRT-PCR.
| Gene name | Forward primer (5'to 3') | Reverse primer (5'to3') |
| --- | --- | --- |
| OsActin | AGGATTACCATGGCCTCAAGAG | CGCGTATCTTCCCCATGAAG |
| OsNAR2.1 | GTCGTCGAGAAGCGCAAGA | GTCCACTGAAGCTGCGAACTT |
| OsNAR2.2 | CGAGAACAAGAAGAAGAACAAGTG | TGCTGGGCGCTGTAGACAC |
| OsNRT2.1 | CCATCATCCGCGACAACC | TGGCGAGCCTGGAGAAG |
| OsNRT2.2 | TCACCGGCGGCATGGACT | TCGTCTTCGCCTTGTGCT |
| OsNRT2.3a | GCCATCCACAAGATCGGTAG | TGTGGAGCTTCCCGTAGTTG |
| OsNRT2.3b | GCCATCCACAAGATCGGTAG | TGTGGAGCTTCCCGTAGTTG |
| OsNRT2.4 | CCCTTCGTCTGCAAAAGGT | TACCTGGACCCGCTGAAGAA |

## Slide 3
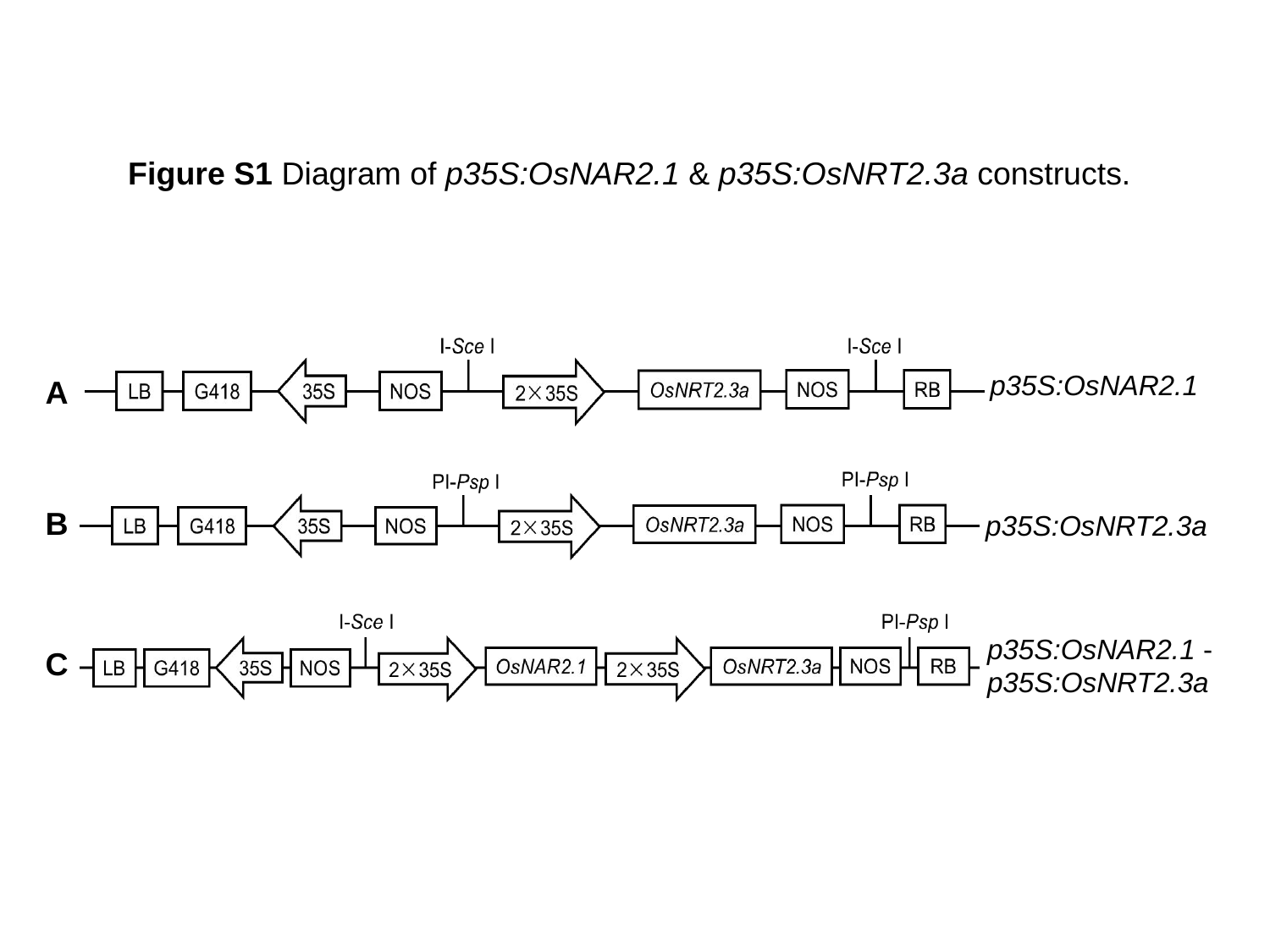

Figure S1 Diagram of p35S:OsNAR2.1 & p35S:OsNRT2.3a constructs.
p35S:OsNAR2.1
A
B
p35S:OsNRT2.3a
p35S:OsNAR2.1 -
p35S:OsNRT2.3a
C

## Slide 4
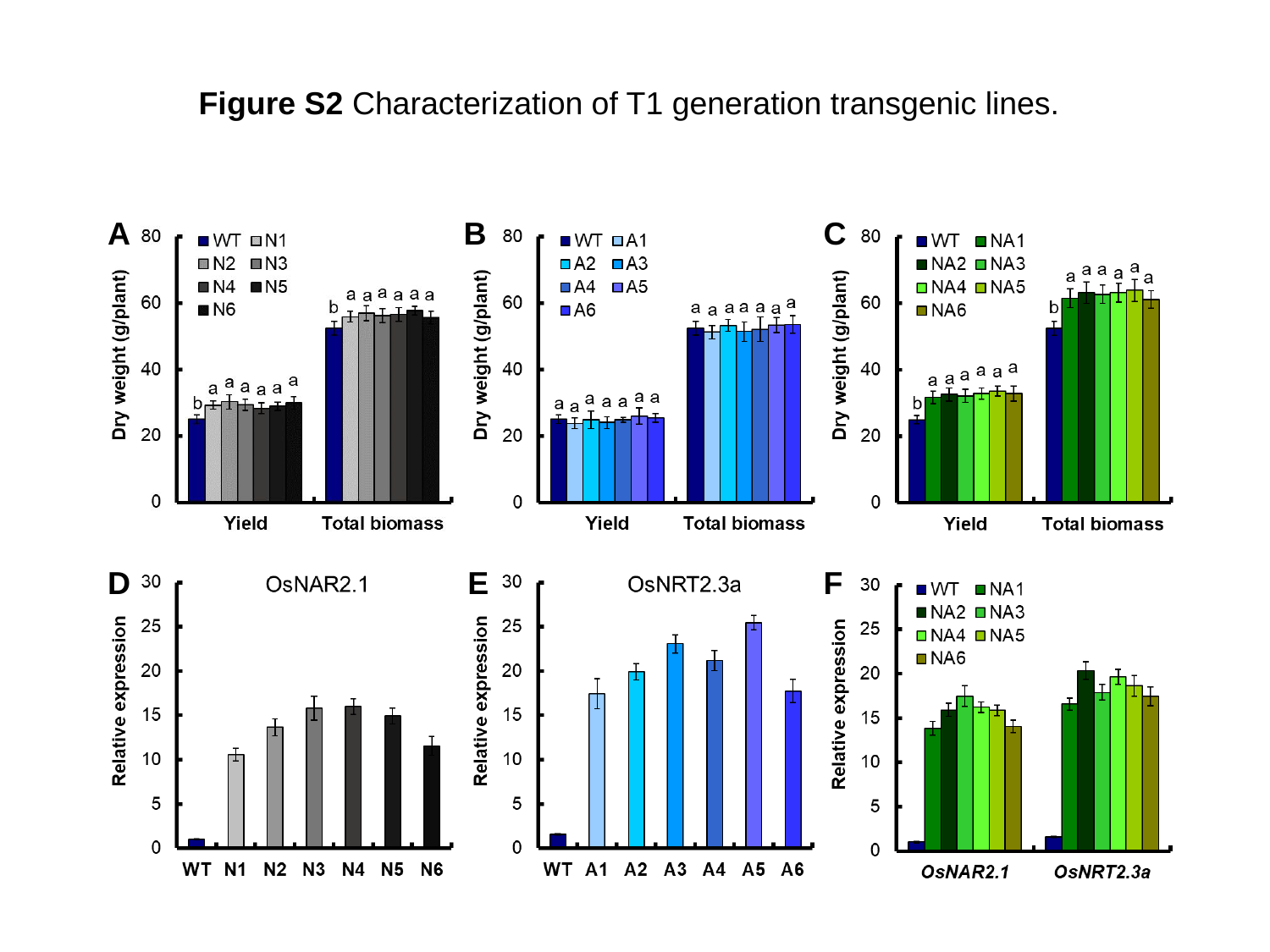

Figure S2 Characterization of T1 generation transgenic lines.
A
B
C
D
E
F

## Slide 5
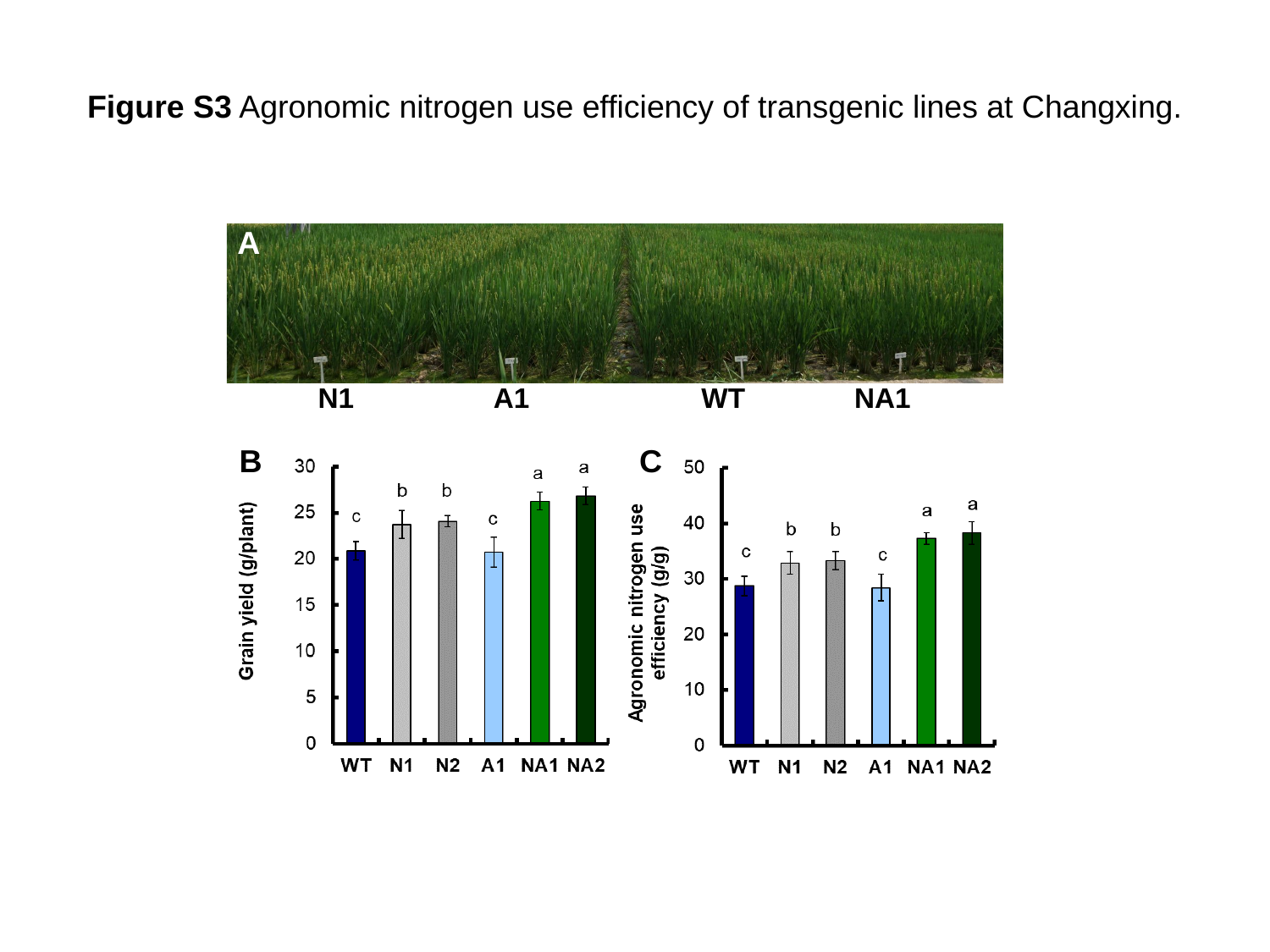

Figure S3 Agronomic nitrogen use efficiency of transgenic lines at Changxing.
A
 N1 A1 WT NA1
B
C

## Slide 6
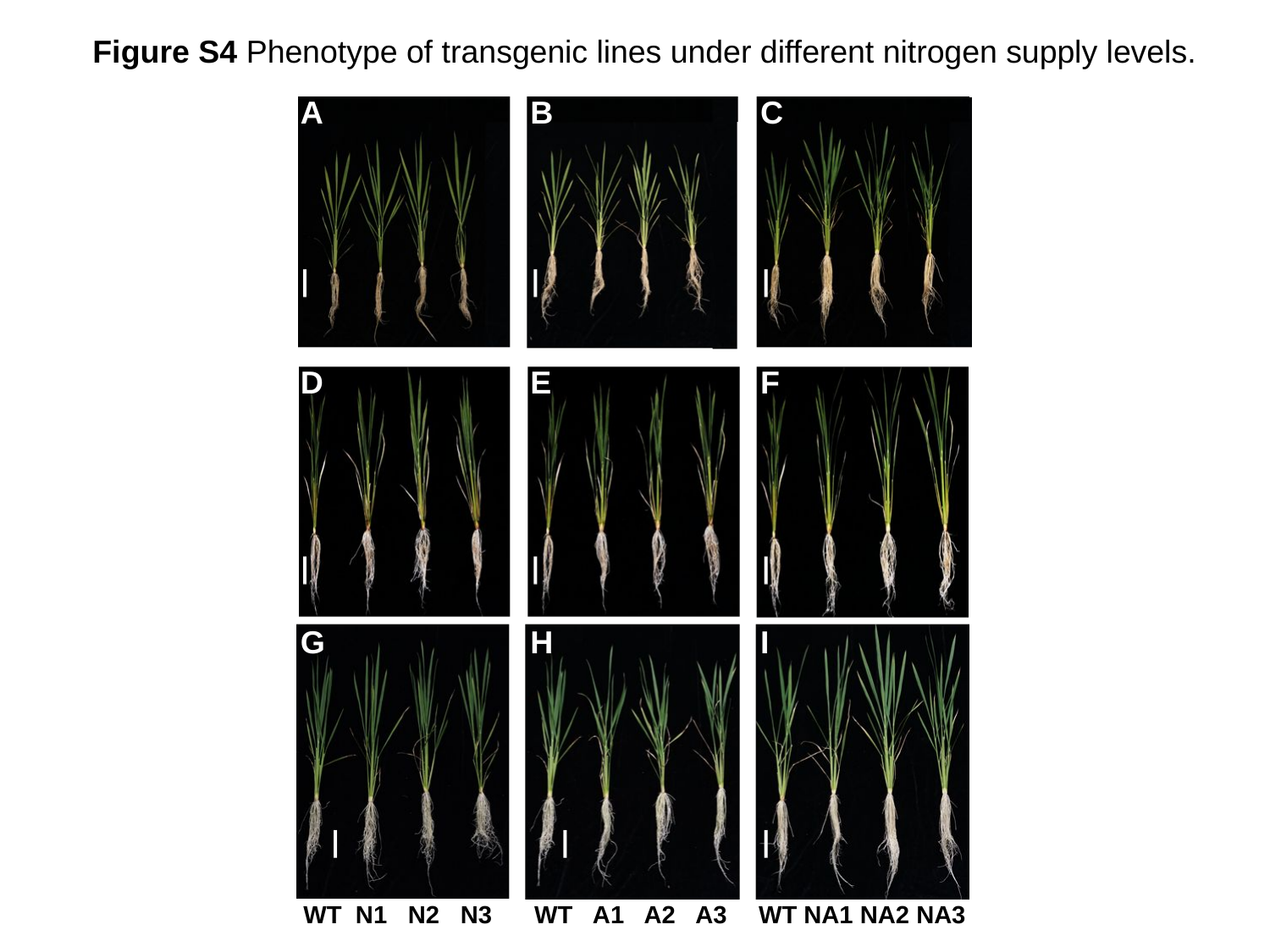

Figure S4 Phenotype of transgenic lines under different nitrogen supply levels.
A
B
C
D
E
F
G
H
I
WT N1 N2 N3
WT A1 A2 A3
WT NA1 NA2 NA3

## Slide 7
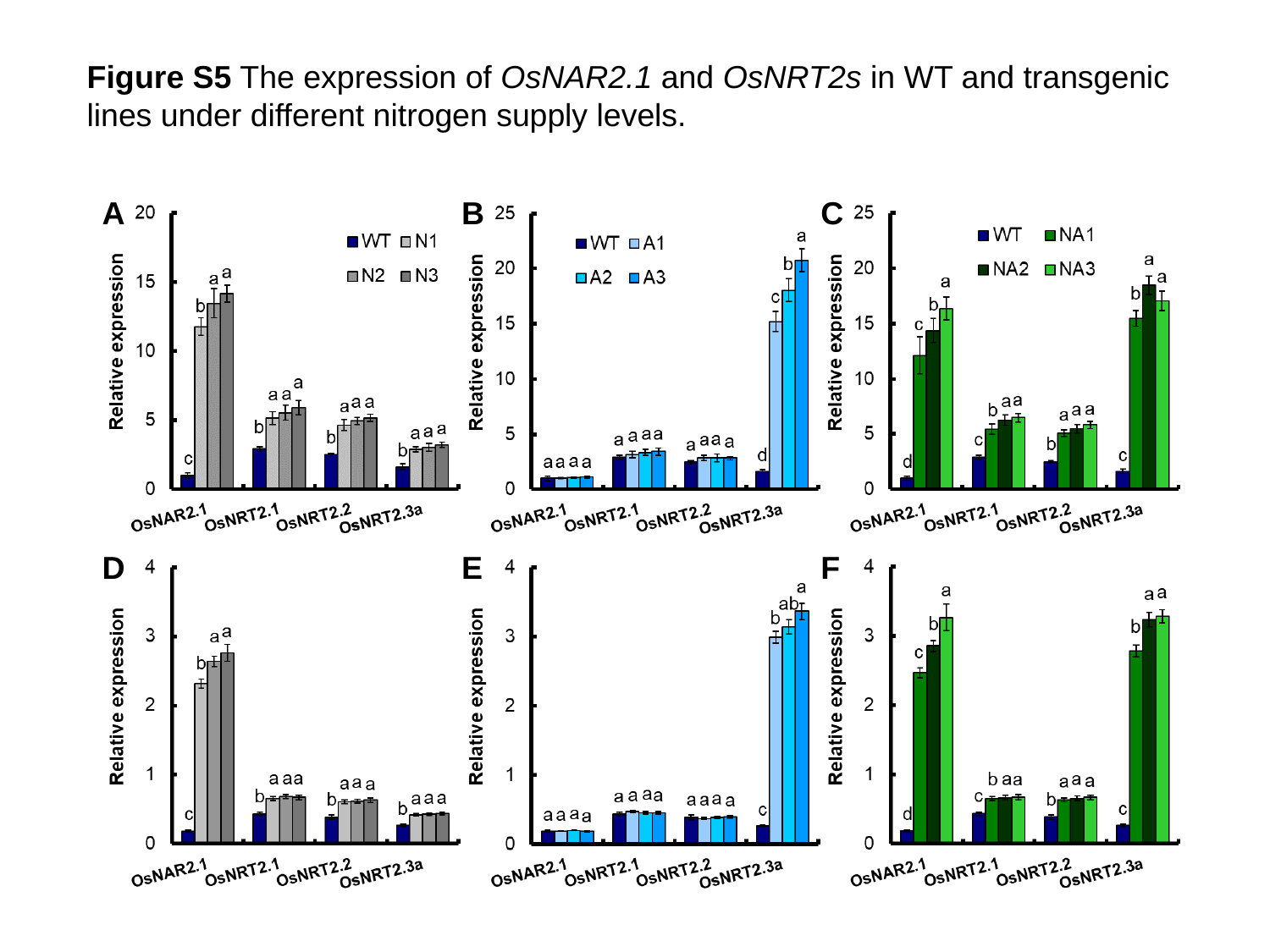

Figure S5 The expression of OsNAR2.1 and OsNRT2s in WT and transgenic lines under different nitrogen supply levels.
A
B
C
D
E
F

## Slide 8
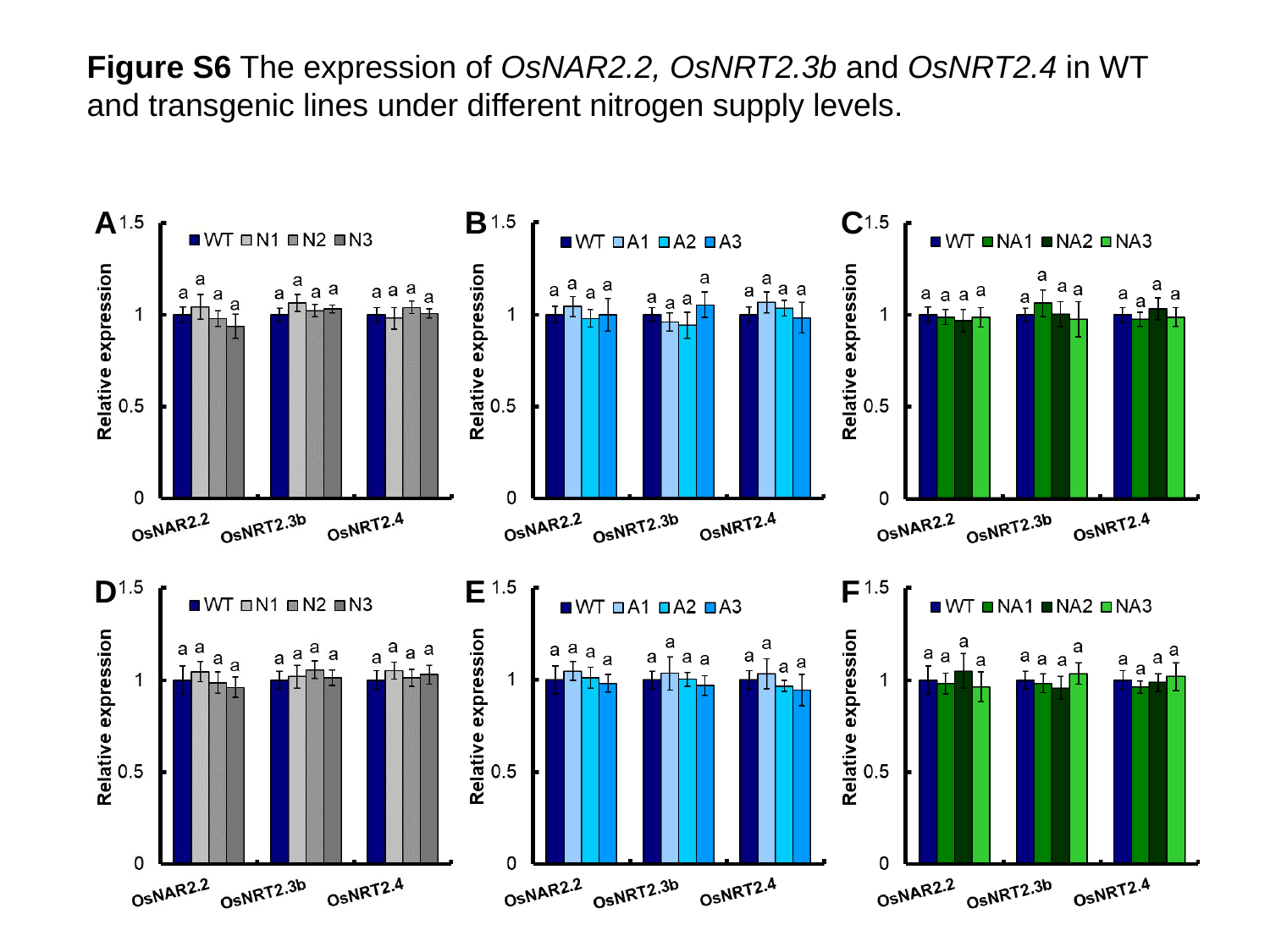

Figure S6 The expression of OsNAR2.2, OsNRT2.3b and OsNRT2.4 in WT and transgenic lines under different nitrogen supply levels.
A
B
C
D
E
F
